# Unusual Case of an Infant with Urinary Tract Infection Presenting as Cholestatic Jaundice

**DOI:** 10.1155/2018/9074245

**Published:** 2018-10-24

**Authors:** Rahaf Niazi, Bashaer Baharoon, Afnan Neyas, Meshari Alaifan, Osama Safdar

**Affiliations:** ^1^Faculty of Medicine, King Abdulaziz University, Jeddah, Saudi Arabia; ^2^Pediatric Department, King Abdulaziz University, Saudi Arabia; ^3^Pediatric Nephrology Center of Excellence, Faculty of Medicine, King Abdulaziz University, Jeddah, Saudi Arabia

## Abstract

Neonatal jaundice is considered one of the most common reasons for admission to the pediatric medical ward. We report a case of a 1-month-old infant who presented with jaundice but no fever or any other signs of systemic illnesses. Laboratory test results revealed high direct hyperbilirubinemia, and urine culture showed a urinary tract infection with* Enterobacter cloacae *as the causative agent. He was admitted to the pediatric medical ward where he was treated with a course of antibiotics for 14 days, and cholestasis resolved completely following a course of antibiotics. We conclude that direct hyperbilirubinemia can be related to urinary tract infection in neonates. It is unusual for urinary tract infection to present clinically and biochemically as cholestatic jaundice.

## 1. Introduction

Indirect hyperbilirubinemia is a known clinical presentation of urinary tract infection- (UTI-) associated urosepsis in infancy. However, cholestatic jaundice is an unusual presentation for UTI. Here, we report the case of a 1-month-old neonate who presented with cholestasis and was discovered to have urinary tract infection. The jaundice resolved completely after completion of a course of antibiotics for UTI.

## 2. Case Presentation

An informed verbal consent was obtained from the parents.

A 1-month-old male neonate with a known antenatal ultrasound (US) diagnosis of fused horseshoe kidneys and bilateral renal hydronephrosis presented in the outpatient clinic with a history of skin jaundice since he was 1 week old. His mother reported a history of passing dark urine and pales tools. The mother also noticed that he was passing a smaller amount of urine and his abdomen was distended. Antenatally, the mother was free from any medical complications during pregnancy; the neonate was delivered by spontaneous vaginal delivery, with a birth weight of 3 Kg

Urinary ultrasound after delivery revealed fused horseshoe kidneys and mild left hydronephrosis. Micturition cystourethrogram (MCUG) was performed, which showed no evidence of posterior urethral valve or vesicoureteral reflux. There was positive consanguinity but no family history of a similar condition or liver disease. He was transferred to the pediatric medical ward for further investigations and management.

Examination upon admission revealed that he had deep jaundice but was not pale. The anterior fontanelle was normally opened, with no dysmorphic features. His vitals were as follows: HR, 104 b/min; RR, 44 cycle/min; blood pressure, 95/50 mmHg; temperature, 36.5 C; and** capillary blood glucose 58 mg/dl** with oxygen saturation 100% in room air. His weight was 3 kg, height 52 cm, and head circumference 35 cm.** He looks dehydrated with dry mucous membrane**. His abdomen was slightly distended and the liver was palpable 2 cm below the costal margin. Other systemic reviews were unremarkable.

Investigation showed elevated white blood cell count 21,000 cell/cumm with 55% polymorphs and 35% lymphocytes, hemoglobin 9.5 g/dl reticulocyte was 3.32%, LDH 180 units/L, platelets 356/cumm, C-reactive protein 50 mg/l, serum total bilirubin 17.78 mg/dl, direct serum bilirubin 15.16 mg/dl, indirect serum bilirubin mg/dL 2.62, serum aspartate transferase 172 IU/L, serum alanine transaminase 162 IU/L, serum gamma-glutamyl transferase (GGTP) 252 IU/L, total serum proteins 5.2 g/dl, serum albumin 2.6 g/dl, and serum sodium 123 mmol/L and** serum creatinine was within normal 0.4 mg/dl**. Urine analysis showed presence of nitrate and leukocyte esterase 500 with WBC 65/hpf. Urine culture showed* Enterobacter cloacae*. Blood culture revealed no growth. TSH was 1.99 mIU/mL. TORCH titers revealed high IgG levels of rubella and cytomegalovirus.

Abdominal US revealed a contracted gallbladder and right ectopic fused kidneys. There was mild hydronephrosis in the left kidney and no hydronephrosis in the right kidney. Both kidneys showed normal corticomedullary differentiation. The urinary bladder showed a thick wall with a turbid content, consistent with cystitis.

Abdominal plain radiography revealed a paucity of the bowel gas in the right side. There was no abnormal bowel loop dilatation. Air within the rectum was noted, without pneumoperitoneum or abnormal calcification.

The patient was started on IV fluid on admission. Then normal saline 3% was started with maintenance D5 NSS to correct his** depletional **hyponatremia and IV antibiotic for 14 days based on the sensitivity pattern. He also received packed red blood cell transfusion for 3 hours due to a drop in his hemoglobin to 5.8 g/dl** sepsis and frequent blood sampling**. IV Vitamin K 5 g and oral ursodeoxycholic acid were administered. Repeated urine culture showed no growth and UTI resolved jaundice completely. He started passing stool freely and was discharged, with regular follow-up.

## 3. Discussion

Neonatal jaundice is a common problem in infancy. It is seen in 60% of full-term and 80% of preterm newborns [1]. Most cases are due to increase in direct fraction of bilirubin, while only 0.04% to 0.2% are cholestatic jaundice [2]. Physiological hyperbilirubinemia is considered the most common cause for jaundice after the first day of life, accounting for 53.9% of cases [3]. Among breast-fed infants, 15% experience some sort of jaundice for more than 3 weeks [4]. However, only few infants with neonatal jaundice are found to have pathological causes, including metabolic and endocrine diseases, underlying hemolytic causes, congenital deficiency of liver enzymes, and bacteremia or sepsis [1]. The latter may cause increased direct fraction of bilirubin in infancy after first week of life, with UTI being the most common cause [5]. Gram-negative organisms were isolated from most cases of sepsis-related jaundice, of which* E. coli *was the most common causative agent [6]. Infants with structural abnormalities in the urinary tract are more susceptible to hyperbilirubinemia associated with UTI. These findings were especially related to hydronephrosis and vesicoureteral reflux, which are consistent with our case report [7, 8].

Some studies have found that hyperbilirubinemia could be the first and only manifestation of neonatal sepsis and UTI [9]. Like in our patient, this was a case of a 2-month-old neonate reported in Turkey in 2017. He presented with jaundice 1 week after birth and urine culture was positive for* Klebsiella pneumonia, *but no other signs of sepsis or urinary tract infection, which is uncommon. However, his jaundice resolved completely after treating his UTI [10]. Another case was reported in an 8-year-old girl who presented with jaundice and UTI due to* E. coli*. Jaundice also resolved completely after treating the UTI [11].

Infants with UTI usually have an increased indirect fraction of bilirubin [11, 12]. This might be due to hemolysins toxins secreted by certain strains of gram-negative bacteria and increased RBC fragility, which eventually causes hemolysis and unconjugated hyperbilirubinemia [13].

However, in our case and the other case reports mentioned above, there was an increase in the direct fraction of bilirubin, which is defined as a conjugated bilirubin concentration of more than 2 mg/dL or more than 20% of total bilirubin [14]. Several mechanisms have been suggested to explain cholestatic jaundice in a setting of UTI. Endotoxins secreted by gram-negative organisms are thought to be the main cause of UTI-related hyperbilirubinemia [15]. A marked decrease in multidrug resistance-associated protein 2, an ATP-dependent transporter involved in the bile- and salt-independent bile export system, under oxidative stress caused by lipopolysaccharide (LPS) endotoxins from bacterial outer membrane, has been reported [15–17]. This causes bile stasis due to impaired excretion and indirectly damages hepatocytes [13].

Another suggested mechanism is direct hepatocellular damage caused by invasion of gram- negative bacteria during an episode of bacteremia. However, the latter mechanism is not reliable as cholestatic syndrome was documented even in the absence of bacteremia [18]. Moreover, LPS released in the blood stream from gram-negative organisms causes suppression of the inner circular muscles of the intestinal wall. This is thought be the cause of sepsis-associated ileus and could explain constipation in our case [5, 19].

In conclusion, UTI is major clinical problem in all children as it is considered the most common cause of febrile illness and is reported in around one-third of bacterial infections in the pediatric age group, especially during infancy [11]. A well-timed management of UTI may prevent many complications, such as hypertension, ESRD, urosepsis, and proteinuria [20]. Although screening for UTI in asymptomatic neonates presenting with only jaundice is still controversial, a strong suspicion of sepsis and serious systemic infection should never be overlooked.
